# Patient-Specific Deep Architectural Model for ECG Classification

**DOI:** 10.1155/2017/4108720

**Published:** 2017-05-07

**Authors:** Kan Luo, Jianqing Li, Zhigang Wang, Alfred Cuschieri

**Affiliations:** ^1^School of Information Science and Engineering, FuJian University of Technology, Xueyuan Road 3, Fuzhou 350118, China; ^2^School of Instrument Science and Engineering, Southeast University, Sipailou 2, Nanjing 210096, China; ^3^Institute for Medical Science and Technology, University of Dundee, Dundee DD2 1FD, UK; ^4^School of Basic Medical Sciences, Nanjing Medical University, Longmian Avenue 101, Nanjing 211166, China

## Abstract

Heartbeat classification is a crucial step for arrhythmia diagnosis during electrocardiographic (ECG) analysis. The new scenario of wireless body sensor network- (WBSN-) enabled ECG monitoring puts forward a higher-level demand for this traditional ECG analysis task. Previously reported methods mainly addressed this requirement with the applications of a shallow structured classifier and expert-designed features. In this study, modified frequency slice wavelet transform (MFSWT) was firstly employed to produce the time-frequency image for heartbeat signal. Then the deep learning (DL) method was performed for the heartbeat classification. Here, we proposed a novel model incorporating automatic feature abstraction and a deep neural network (DNN) classifier. Features were automatically abstracted by the stacked denoising auto-encoder (SDA) from the transferred time-frequency image. DNN classifier was constructed by an encoder layer of SDA and a softmax layer. In addition, a deterministic patient-specific heartbeat classifier was achieved by fine-tuning on heartbeat samples, which included a small subset of individual samples. The performance of the proposed model was evaluated on the MIT-BIH arrhythmia database. Results showed that an overall accuracy of 97.5% was achieved using the proposed model, confirming that the proposed DNN model is a powerful tool for heartbeat pattern recognition.

## 1. Introduction

Cardiovascular diseases (CVDs) remain the leading cause of noncommunicable deaths worldwide. According to the latest World Health Organization (WHO) report, about 17.5 million people died from CVDs in 2012, accounting for 30% of all global deaths. The incidence of CVD deaths is predicted to rise to 23 million by 2030 [1]. Furthermore, the costs for CVD-related treatment including medication are substantial. The CVD-related cost in the low- and middle-income countries over the period 2011–2025 is estimated approximately 3.8 trillion U.S. dollars [2]. Many of these deaths and associated economic losses can be avoided by early detection and monitoring of patients' cardiac function. Electrocardiogram (ECG) is the standard and most efficient tool for CVD diagnosis [3], which captures the electrical activity of the heart from a human body surface, providing important information on cardiac functional abnormalities. The recent introduction of technology for the wireless body sensor network- (WBSN-) enabled ECG has attracted the attention of both industry and academic researchers. WBSN-enabled ECG biosensors are seamlessly integrated into wearable fabric vest and can provide real-time continuous 7/24 monitoring and cardiac arrhythmia detection [4, 5]. This wearable WBSN-enabled ECG has the essential need for more efficient and robust data analysis methods for long monitoring of individual patients to ensure timely medical treatment or intervention. However, there are still some challenges in WBSN-enabled ECG signal analysis, particularly for the automatic detection of life-threatening arrhythmias [6].

Traditional methods exited risk of improper manual feature selection and limiting complex classification ability. There have been several reports on heartbeat classification [7–14]. Ince et al. [7] proposed an artificial neural network- (ANN-) based automated heartbeat classification model with morphological wavelet transform features, which achieved highly accurate heartbeat classification. Jiang and Kong [8] used a block-based neural network model with Hermite transform coefficients and the selected temporal features for personalized ECG signal classification. Ye et al. [9] used morphological features and RR interval information in a support vector machine classifier for heartbeat classification. Alvarado et al. [10] proposed a novel compression sampler for feature extraction of ECG beats and then utilized linear discriminant analysis (LDA) for their classification. Chazal and Reilly [11] also used LDA as a classifier of differential temporal features including heartbeat morphology, heartbeat intervals, and R-R intervals. There are more studies on feature and classifier model [12–14]. Typically, the traditional classification models contain two layers at most, restricting their ability for complex classification tasks. Meanwhile, most of these models need manually designed features. Even the best classifier model will yield poor performance if important features are not selected. Additionally, most reported works focus on establishing common interpatient models for heartbeat classification. These methods were not using samples from the same patient for model training and testing [14]. However, WBSN-enabled ECG monitoring emphasized personalized heart status care; relatively common and patient-specific samples will help training a good performance model in individual heartbeat classification [15, 16]. Patient-specific method is more suitable in a WBSN-enabled ECG monitoring scenario.

Deep learning (DL) is an ideal and potential approach for the heartbeat classification of WBSN-enabled ECG, which can further improve classification performance. As a new research area of machine learning, DL has progressed rapidly since 2006 [17–19]. DL is based on algorithms for learning multiple levels of representation for the modeling complex relationship between data sets. Specifically, it is recognized as an effective method of abstracting hierarchical representation from unlabeled data; since higher-level features are defined by lower-level ones, the hierarchical feature representation of DL is referred to as “deep architecture” [20]. DL models by virtue of their multiple levels and nonlinear information processing provide much more efficient representations of complex functions, resulting in improved performance compared to shallow models [21]. Several studies have confirmed that deep architectural models exhibit excellent performance beating the existing traditional methods in challenge classification tasks [15, 22, 23]. However, there are still some aspects that need to be further studied when DL methods are used in traditional ECG analysis, such as the parameter of layers, size of neurons, and use of tanning samples.

Motivated by these challenges, we proposed a patient-specific heartbeat classification framework using time-frequency representation and a DL architectural model. Considering time-frequency technology is a powerful tool for characterizing the biosignals [24], and some of DL frameworks, such as stacked auto-encoder, convolutional neural networks (CNNs), and deep belief nets (DBNs) [22, 23, 25, 26], can be used to analyze ECG signal, while heartbeat time-frequency spectrograms are seen as images. A modified frequency slice wavelet transform (MFSWT) was used to generate time-frequency representation of the heartbeat signal. Stacked denoising auto-encoder (SDA) model was chosen as the DL architectural model in our works. A SDA was pretrained by unlabeled MFSWT time-frequency spectrograms. Subsequently, a deep neural network (DNN) model was initialized by weights and bases of the trained SDA and was followed by two levels of fine-tuning. Particularly, after the second fine-tuning stage by using individual annotated heartbeat samples, the patient-specific DNN classifier was obtained. Validation of the proposed heartbeat classification method was performed on MIT-BIH arrhythmia database.

## 2. Data Description

MIT-BIH arrhythmia database [27] was selected as the data source, which is the most commonly used database for research in ECG signal processing. It consists of 48 annotated, 30 min ambulatory ECG records from 2 leads (II and modified V1, V2, V3, V4, or V5 leads) obtained from 47 subjects and sampled at 360 Hz per channel. Since lead II ECG is commonly used in ambulatory or WBSN-based ECG applications, these channel data were used in the current study.

The five heartbeat classes defined in the American National Standards Institute (ANSI) for the Advancement of Medical Instrumentation (AAMI) standard (IEC 60601-2-47:2012) [28] are (i) normal beat (N), (ii) supraventricular ectopic beat (SVEB or S), (iii) ventricular ectopic beat (VEB or V), (iv) fusion beat (F), and (v) paced beats or unknown beat Q. However, according to the annotation file from PhysioNet (http://www.physionet.org/), there are 15 beat types in MIT-BIH database.  Table 1 shows the group method for mapping the MIT-BIH heartbeat classes into AAMI classes.

ANSI/AAMI EC57:2012 recommends exclusion of records containing paced beat records (numbers 102, 104, 107, and 217) for classifiers' evaluation. The number of 33 unclassified beats is less than 0.03% of the whole data samples, which can lead to model overfitting. Thus, in this study, Q type includes paced beats and unclassified beats were excluded. Following these exclusions, the heartbeat data samples were regrouped into four types (N, S, V, and F) according to the AAMI standard. Thus, the remaining 44 nonpacemaker records without the unclassified beats were divided into equal training and testing sets [10]. The training set consisted of record numbers 101, 106, 108, 109, 112, 114, 115, 116, 118, 119, 122, 124, 201, 203, 205, 207, 208, 209, 215, 220, 223, and 230; and the testing set consisted of record numbers 100, 103, 105, 111, 113, 117, 121, 123, 200, 202, 210, 212, 213, 214, 219, 221, 222, 228, 231, 232, 233, and 234.

The objective of the proposed framework is to classify heartbeats into N, S, V, and F classes. Clinically, supraventricular ectopic beats (SVEB) and ventricular ectopic beats (VEB) are two critically abnormal and serious heartbeats, and the performance of the classifiers also was elevated by testing S and V heartbeat classification [9, 10, 15].

## 3. DL Architectural Model-Based Heartbeat Classification

**Figure 1:**
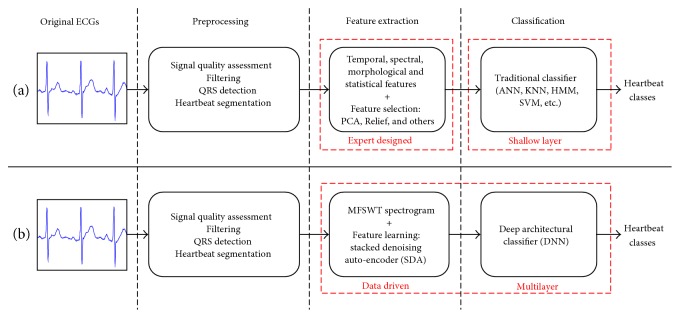
Schematic illustration of two heartbeat classification frameworks: (a) traditional framework and (b) the framework of proposed DL architectural model with time-frequency representation.

The comparison between traditional and proposed heartbeat classification frameworks is shown in Figure 1. Both include three steps: preprocessing, feature extraction, and classification.

The key differences, which distinguish the new framework, are as follows:

- The use of MFSWT to generate time-frequency spectrogram for using deep learning methods
- Adoption of stacked denoising auto-encoder (SDA) for automatic abstraction of features from MFSWT spectrogram (instead of human experts), to avoid the associated risk of improper manual feature selection
- Integration of data-driven feature extraction and DL architectural classification into a single learning framework for improved heartbeat classification.

### 3.1. Preprocessing of ECG Signal

**Figure 2:**
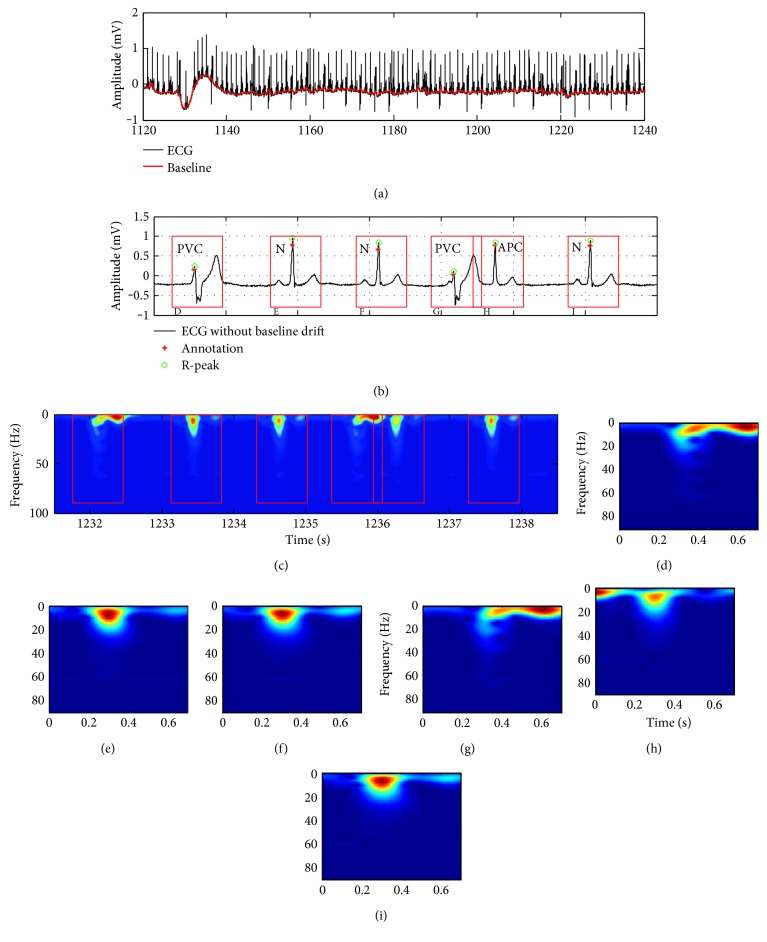
ECG preprocessing, sample heartbeat waveform from number 201 record lead II, including normal (N), premature ventricular contraction (PVC), and atrial premature contraction (APC) AAMI heartbeat classes. (a) Baseline drift elimination; (b) QRS-complex detection; (c) MFSWT spectrogram corresponding to the waveform in (b); (d)~(i) MFSWT spectrograms corresponding to each segmented heartbeat by 700 ms windows in (b). In the absence of other special instructions, all spectrograms were normalized to [0, 1].

Preprocessing of ECG signal includes signal quality assessment (SQA) [29], denoising, QRS detection, heartbeat segmentation, and calculation of time-frequency spectrogram. Since the present study concerns the illustration of DL-based model for heartbeat classification, the SQA step was omitted, except for the removal of low signal quality heartbeats and their pre- and after beats from the data set. Power and high-frequency noise and baseline drift were eliminated by using two median filters with window sizes of 200 ms and 600 ms [10] as shown in Figure 2(a). Although many algorithms are used for QRS detection [30], the derivative-based algorithm with a characteristic steep slope of the QRS complex was chosen to detect R-peaks in view of its high accuracy.  Figure 2(b) provides details of the comparison of the detected R-peaks and the annotation points where all six R-peaks can be identified. 700 ms windows (red boxes in Figure 2), centered at the detected R-peaks (300 ms before and 400 ms after), were used to segment each heartbeat. As shown in Figures 2(b) and 2(c), the normal, premature ventricular contraction (PVC), and atrial premature contraction (APC) beats are segmented, and the corresponding MFSWT spectrograms were shown in Figures 2(b)~2(i).

### 3.2. Modified Frequency Slice Wavelet Transform

Frequency slice wavelet transform (FSWT) is essentially an extension of the short-time Fourier transform in frequency domain [31]. FSWT achieves good performance in transient vibration response analysis and damping modal identification [32]. However, low-frequency biosignals are not well represented by original FSWT due to its defined window size of frequency slice function (FSF) changes sharply in low-frequency area. To accurately locate the components of heartbeat signal in time-frequency plane, “modified frequency slice wavelet transform (MFSWT)” was proposed. MFSWT follows the rules of producing time-frequency representation from the frequency domain but incorporating a set of bound signal-adaptive FSFs which serve as a set of dynamic frequency filters, which can well represent signal in time-frequency domain.

Assume that $\hat{f}\left(u\right)$ is Fourier transform of *f*(*t*). The MFSWT is expressed in frequency domain as

(1) $$\begin{array}{c} W_{f}\left(t,\omega \right)=\frac{1}{2\pi }\int_{-\infty }^{+\infty }\hat{f}\left(u\right){\hat{p}}^{*}\left(\frac{u-\omega }{q\left(\hat{f}\left(u\right)\right)}\right)e^{iut}du, \end{array}$$

where *t* and *ω* are observed time and frequency, respectively. “∗” represents the conjugation operator. $\hat{p}\left(x\right)=e^{-x^{2}/2}$ is selected as the FSF in (1), and $\hat{p}\left(0\right)=1$. The shape of FSF is like an inverted bell. *q* is defined as a scale function of $\hat{f}\left(u\right)$ and enables the transform with signal-adaptive property.

(2) $$\begin{array}{c} q=\delta +sign\left(\nabla \left|\hat{f}\left(u\right)\right|\right), \end{array}$$

where *δ* is the frequency which corresponds to the maximum $\left|\hat{f}\left(u\right)\right|$. ∇(·) is differential operators, and sign(·) means signum function, which returns 1 if the input is greater than zero, 0 if it is zero, or −1 if it is less than zero. According to (2), *q* changes slowly with $\left|\hat{f}\left(u\right)\right|$ and generates FSFs as a function of $\left|\hat{f}\left(u\right)\right|$. As dynamic frequency filters, FSFs were used to estimate the energy distribution of different frequency bands. Similar to the scale used for different size objects in microscopy, narrow window size of FSFs corresponds to the small values of $\left|\hat{f}\left(u\right)\right|$ and wide window size of FSFs corresponds to the large values of $\left|\hat{f}\left(u\right)\right|$. Due to the effect of the adaptive FSF, energy of signal components with large $\left|\hat{f}\left(u\right)\right|$ can be reinforced in time-frequency spectrogram. Taking advantages of the slowly changing FSFs and energy enhancement of frequency filtering, MFSWT achieves accurate time-frequency representation of the heartbeat signal.

FSFs in MFSWT meet $\hat{p}\left(0\right)=1$ according to the proof in [32]. The reversed MFSWT can be expressed by

(3) $$\begin{array}{c} f\left(t\right)=\frac{1}{2\pi }\int_{-\infty }^{+\infty }\int_{-\infty }^{+\infty }W_{f}\left(\tau ,\omega \right)e^{i\omega \left(t-\tau \right)}d\tau d\omega . \end{array}$$

**Figure 3:**
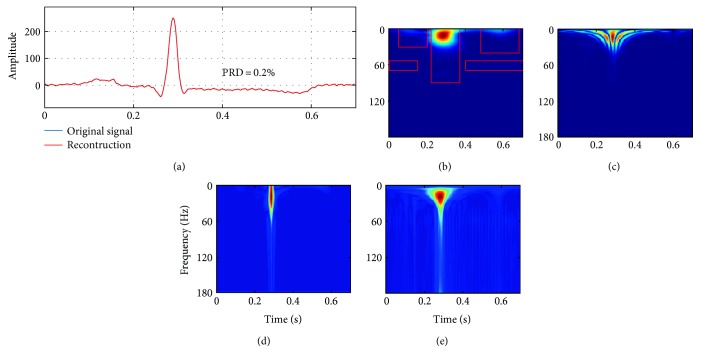
Heartbeat signals and corresponded spectrograms: (a) original heartbeat and reconstructed heartbeat; (b) MFSWT; (c) CWT @ mexh wavelet (d) WVD; and (e) FSWT @ *κ* = 4. All spectrograms were normalized to [0, 1].

In this study, MFSWT is used as a tool to generate heartbeat spectrogram for SDA feature extraction.  Figure 3 shows an example. The comparison between the original and the reconstructed heartbeat signal is shown in Figure 3(a). Percentage root-mean-square difference (PRD) equal to zero indicates that signal can be exactly reconstructed by the reversed MFSWT from the spectrogram. Heartbeat time-frequency spectrograms of MFSWT, Wigner-Ville distribution (WVD), continuous wavelet transform (CWT), and FSWT are shown in Figures 3(b)~3(e), respectively. As outlined in Figure 3(b), accurate locations of P-, QRS-, and T-waves and power noise components in time-frequency spectrogram, which correspond well to the signal in the time domain, were achieved by using the MFSWT. In comparison to WVD, CWT, and FSWT, the spectrogram of MFSWT has better interpretability. Additionally, without troublesome parameter selection, MFSWT is easier to use than other methods. The goal of machine learning is to replace humans for pattern recognition, considering MFSWT spectrograms are more readily accepted by clinicians, which are adopted as time-frequency images for DL classification in the present work.

### 3.3. Stacked Denoising Auto-Encoder

Auto-encoder (AE) is the basic unit of stacked denoising auto-encoder, which can capture the maximum possible information contained in a given sample, while minimizing the reconstruction error rate. A basic encoder is a function that takes an input *V* ∈ *R*^*d*_*V*_^ to a hidden representation *h* ∈ *R*^*d*_*h*_^, which can be stated as

(4) $$\begin{array}{c} h=s_{AE}\left(WV+b\right), \end{array}$$

where *W* is a *d*_*V*_ × *d*_*h*_ weight matrix, *b* ∈ *R*^*d*_*h*_^ is a bias, and *s*_AE_ is a nonlinear logistic sigmoid activation function *s*(*x*) = 1/(1 + *e*^−*x*^).

The decoder maps the output of the hidden layer *h* back to the reconstruction $\hat{V}$ by a similar transformation.

(5) $$\begin{array}{c} \hat{V}=s_{DC}\left(W^{\prime }h+b^{\prime }\right), \end{array}$$

where *s*_DC_ is also a logistic sigmoid activation function and $W_{d_{h}\times d_{\hat{V}}}^{\prime }$ and $b^{\prime }\in R^{d_{\hat{V}}}$ are two parameters of decoder. Let *W*′ = *W*^*T*^ be referred to as tied weights.

The parameters of AE are optimized if the average reconstruction error is minimized, which corresponds to minimize the following objective function:

(6) $$\begin{array}{c} O\left(\left\{W,b,b^{\prime }\right\}\right)=\sum_{V\in D_{n}}L\left(V,\hat{V}\right), \end{array}$$

where *L* is a reconstruction error. The function of cross-entropy loss can be used as *L* if input samples *D*_*n*_ for training are in [0, 1].

(7) $$\begin{array}{c} L\left(V,\hat{V}\right)=-\sum_{k=1}^{d_{V}}\left[V_{k}\log\left({\hat{V}}_{k}\right)+\left(1-V_{k}\right)\log\left(1-{\hat{V}}_{k}\right)\right]. \end{array}$$

For robust feature extraction, Vincent et al. [26] proposed denoising auto-encoders (DAE). DAE is trained to reconstruct the input from a corrupted version of the input. Thus, the model has an antinoise property. The objective function of DAE can be written as in (8), which can be optimized by the stochastic gradient descent method [21].

(8) $$\begin{array}{c} O\left(\left\{W,b,b^{\prime }\right\}\right)=\sum_{V\in D_{n}}E_{\tilde{V}\sim q\left(\tilde{V}\;|\;V\right)}\left[L\left(V,\hat{V}\right)\right]. \end{array}$$

**Figure 4:**
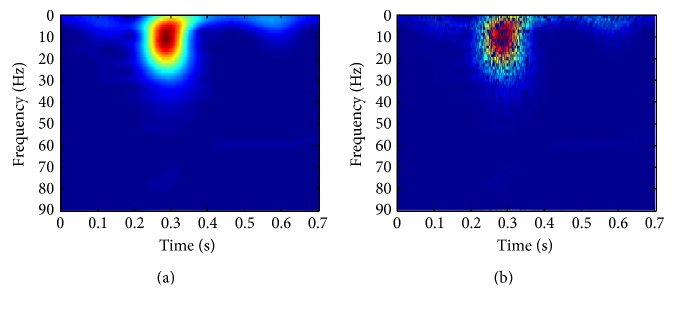
Stochastic corruption process in SDA model training. (a) Original spectrogram. (b) Result of stochastic corruption process (*P* = 0.5).

In (8), *E* is the expectation, and the corrupted version $\tilde{V}$ of *V* produces $q\left(\tilde{V}|V\right)$ by a corruption process. Stochastic corruption process, which randomly sets a fraction *P* of inputs to zero, is used as the corruption process in this work, and an example is shown in Figure 4(). The parameter *P* controls the degree of regulation.

**Figure 5:**
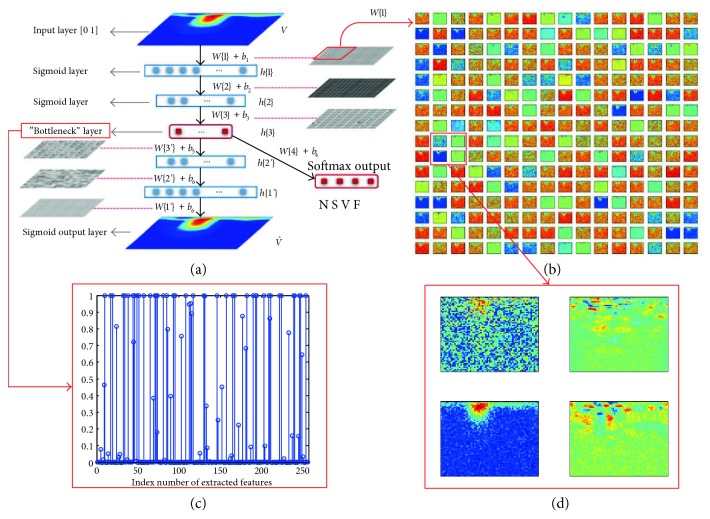
Schematic view of SDA for feature learning and DNN-based classification: (a) three layers of SDA and four layers of DNN; (b) part of the first layer weights of unsupervised trained SDA model; (c) extracted features of number 100 record first heartbeat; and (d) details of the weights marked with a red box.

SDA is achieved by stacking multiple DAEs with their corresponding decoders. Here, the SDA was used for primary feature extraction and initialization of deep neural network weights. The schematic view of a three-layered SDA is shown in Figure 5. Each layer of SDA is a DAE, and unsupervised layer-by-layer training minimizes the reconstruction error of each DAE.  Figure 5(b) shows part of first layer weightings of unsupervised trained SDA model. Details of weights marked with a red box in Figure 5(b) are shown in Figure 5(d), which demonstrates that spectral features of heartbeat are captured by the trained SDA model and are stored as the weights. The extracted features of number 100 record first heartbeat are shown in Figure 5(c), In this case, the number of final abstracted features is 256, and the output of the bottleneck layer is sparsity, which helps the subsequent discriminant classification.

### 3.4. DNN Classifier

Since heartbeat classification is a multiple output task, a softmax regression layer with “N, S, V, and F” output is added on top of the bottleneck layer as shown in Figure 5(a). Then, the SDA encoder and the softmax layer are combined to form a DNN classifier. *W*{i}, *b*_*i*_, and h{i} are the weights, bases, and outputs of each hidden layers, respectively. The heartbeat class can be identified when the final hidden layer output feeds into the last softmax layer.

## 4. Patient-Specific DNN Classifier Training

Due to existing interpatient signal variability, different patients' beats of the same class are different; it is difficult to train a common interpatient model, which can perfectly classify heartbeats from other patients. This problem can be overcome by the use of patient-specific technique [7, 8, 11]. Here, the patient-specific approach was adopted in the present study. We used a small beginning part of individual samples in model training to maximize performance in individual heartbeat classification.

**Figure 6:**
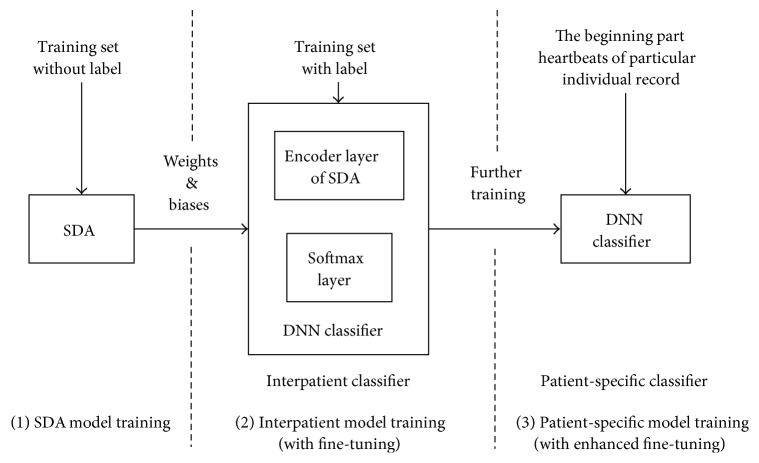
The workflow of patient-specific DNN training.

As shown in Figure 6, the whole patient-specific DNN training consists of three sequential stages: (i) SDA model training, (ii) common interpatient classifier training, and (iii) patient-specific classifier training. The first stage is training the SDA model. Its purpose is to estimate initial parameters of a DNN classifier from the trained SDA. The second stage is referred as fine-tuning [16–18]. After using encoder layers of SDA and a softmax layer forming a DNN classifier, fine-tuning is used to minimize the heartbeat classification prediction error with samples of training set listed in Section 2. After fine-tuning, an interpatient DNN classifier is achieved. In the third stage, newly annotated heartbeats of the testing set (i.e., first 300 beats) are used for further fine-tuning based on the trained interpatient classifier. In this stage, the same algorithm in the second stage is adopted but with different train samples. The program will stop until classification performance has no further improvement or the maximum number of iterations is reached. At this stage, personalized heartbeat DNN classifiers can be generated. The last stage called as enhanced fine-tuning in our work aims to further parameter adjustment of the trained interpatient classifier to address the individual variations.

## 5. Experimental Results and Discussion

Evaluation of the trained classifier by the AAMI standard was done with MIT-BIH arrhythmia database. In preprocessing, all MFSWT spectrograms of each heartbeat were normalized to [0, 1], and records in the database were divided into equal training and testing sets as described previously. The evaluation results were compared to those reported by other systems [7–14].

Four widely used metrics, that is, sensitivity (SE), specificity (SP), positive predictive value (PPV), and accuracy (ACC), were used (and defined next) for the assessment of classification performance:

(9) $$\begin{array}{c} {SE}_{i}=\frac{{TP}_{i}}{\left({TP}_{i}+{FN}_{i}\right)},\\ {SP}_{i}=\frac{{TN}_{i}}{\left({TN}_{i}+{FP}_{i}\right)},\\ {PPV}_{i}=\frac{{TP}_{i}}{\left({TP}_{i}+{FP}_{i}\right)},\\ {ACC}_{i}=\frac{\left({TP}_{i}+{TN}_{i}\right)}{\left({TP}_{i}+{TN}_{i}+{FP}_{i}+{FN}_{i}\right)}, \end{array}$$

where TP_*i*_ (true positive) equals the number of *i*th class heartbeats correctly classified, TN_*i*_ (true negative) is the number of heartbeats not belonging to *i*th class and not classified in the *i*th class, FP_*i*_ (false positive) equals the number of heartbeats erroneously classified into *i*th class, and FN_*i*_ (false negative) equals the number of *i*th class heartbeats classified in a different class. SE_*i*_ and SP_*i*_, respectively, reflect the classifier's sensitivity and specificity in *i*th prediction, and PPV_*i*_ defines the percentage of positive correct predictions. ACC_*i*_ is the ratio between all correctly and incorrectly predicted heartbeats. Since the data set is imbalanced, the geometric mean (*g*-mean) [13], estimated by the geometrical mean of heartbeat class predicted sensitivities, was also selected as a performance measure.

(10) $$\begin{array}{c} g-{\text{mean}}_{x}=\sqrt{x^{+}\cdot x^{-}}, \end{array}$$

where *x*^+^ and *x*^−^ are the predicted SE or PPV of the positive and negative classes, respectively.

**Figure 7:**
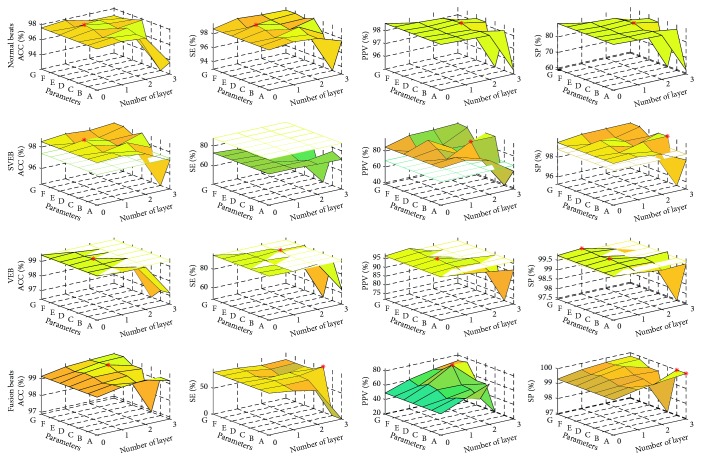
Heartbeat classification results by patient-specific models with different parameters. The best results of SVEB and VEB classification from other works in Table 4 are shown as benchmarks; the best results of the proposed models are marked by asterisks. A~G represent the sizes of the first hidden layer which are 64, 128, 256, 512, 1024, 2048, and 4096, and the number of neurons of the next layer was set as half of the current layer if SDA is a multilayer structure.

Grid searching was used to identify the optimal parameters. The number of layers was changed from 0 to 3. Considering the feature abstract characteristic of SDA, it is a good idea to make the size of the hidden layer output smaller than the input size for each AE or DAE. Seven conditions of the number of neurons in the first hidden layer are A (64), B (128), C (256), D (512), E (1024), F (2048), and G (4096). The number of neurons of next layer was set as half of the current layer if SDA is a multilayer structure. After encoder layers, softmax layer maps the abstracted features to four types of heartbeats. For example, in a 3-layer SDA model, the number of the first layer is 512 and then the numbers of neurons of each layer in DNN model are 512-256-128-4. The beginning 300 beats of test records were used for personalized classifier training. Experimental results were based on the remaining heartbeats of testing sets and shown in Figure 7. The SVEB and VEB classification results from other works are shown in Table 4 as benchmarks in Figure 7, and the best results of our work are marked by asterisks. With proper parameters, the proposed method outperforms the benchmarks in all other measures except SE of SVEB. The experimental results confirm the efficiency of the proposed patient-specific deep architectural framework. However, to maintain good stable performance, the size of neurons can be selected with a narrower range while the number of layers is added. Based on the classification results and generalization risk consideration, it is advised that one hidden layer within 1024~2048 neurons could achieve acceptable heartbeat classification.

**Figure 8:**
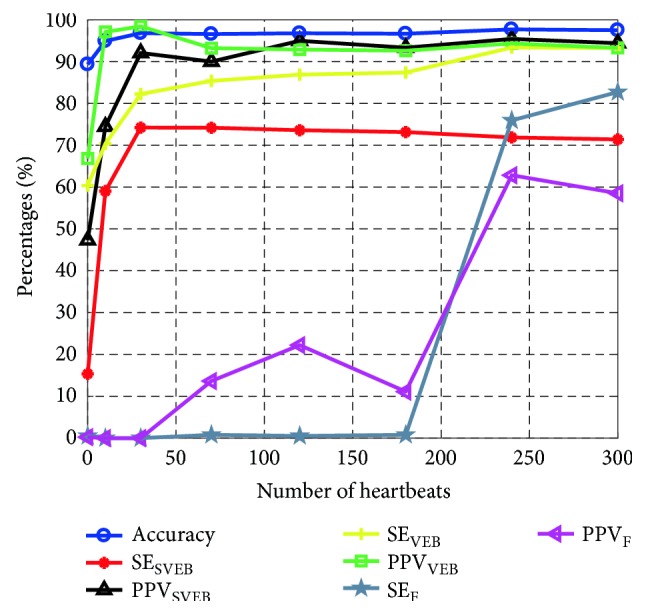
Relationships between classification performance and the number of individual samples were used for model training.

0 to 300 heartbeats of individual samples were used in patient-specific models training to explore the relationship between performance improvement and the added number of personal samples. Experiments were based on a trained interpatient DNN classifier, which includes one encoder layer with 1024 neurons and a softmax layer. The relationship is shown in Figure 8. Accuracy, SE, and PPV increase as the number of individual heartbeats increase. Accuracy and measures of SVEB and VEB become stable when the number of beats exceeds 80. Because major F beats of the testing set are in number 213 record and appear after 2 min, the best SE and PPV of F were achieved in the range of 240~300 beats. The proposed method would produce an interpatient classifier if no individual samples were used in fine-tuning. Confusion matrix of interpatient heartbeat classification (Table 2) demonstrates an 89.3% heartbeat classification accuracy. Although SE and PPV of N reached 95.3% and 93.0%, respectively, the other measure values of S, V, and F are low. From the results, it can infer that the constructed interpatient deep architectural classifier has poor ability to process interpatient signal variability.

The overall classification assessment results obtained by considering each of the four classes (“N, S, V, and F”) are summarized in Table 3 when 300 beats are used. The numbers of correct prediction of N, S, V, and F beats are 37,622, 1143, 2644 and 292, respectively. The overall accuracy of the heartbeat classification reached 97.5%. The results demonstrate that performance of the classifier can be efficiently improved by using relatively small individual samples, the patient-specific classifier can well cope with interpatient variations.

Comparisons of the proposed model and the state-of-the-art methods [7–14] were summarized in Tables 4 and 5, which demonstrate that the proposed model achieves better recognition in patient-specific heartbeat classification scenario, with ACCs of SVEB and VEB rates of 98.8% and 99.1%, respectively. Specifically, a PPV of SVEB of 94.4% indicates that the proposed model has the high-level capability of identifying SVEB. The 71.4% SE of SVEB is superior to most other reported studies (see Table 4). The results presented in Table 4 confirm that the proposed model can satisfactorily identify SVEB and VEB. Evaluation results for all four-class heartbeat recognitions are outlined in Table 5, and classification confusion matrix in Table 3. The results relating to SE and PPV of all types are close to or surpass those obtained with current state-of-the-art methods. Accuracy, *g*_mean_SE_, and *g*_mean_PPV_ reached 97.5%, 85.9%, and 84.4%, respectively. Similarly, using 1-D CNNs for patient-specific ECG classification that also achieved superior performance was reported [15]. These make us have good reasons to believe that deep learning methods have great potential in patient-specific ECG signal analysis.

The reasons for the superior performance of the proposed method are multifactorial. In the first instance, MFSWT transforms ECG signal from time domain to time-frequency domain. The distinguishable differences of heartbeat signals are well preserved in MFSWT spectrogram, facilitating both SDA feature extraction and the following personalized DNN classifier training. Secondly, using data-driven SDA instead of expert human involvement could avoid the improper feature extraction for classification. Thirdly, deep architectural patient-specific classifier improves the accuracy of individual heartbeat prediction. The main limitation of the present study is that it needs extra individual annotated beats. According to the experimental results, patient-specific samples are important to the proposed method. As the results shown in Figure 8, not using individual samples in the model training, the system may entirely or partially fail to classify S, V, and F beats. However, using a few annotated individual beats is possible in clinical practice. Once the patient-specific classifier has been trained, it can continually provide accurate heartbeat classification services for individual patients in WBSN-enabled long-term automatic ECG monitoring scenario. Except for individual training samples, the computation may limit the usage of the proposed method. The personalized DNN model training requires the extensive computation (~1 hour @ Intel i7 4720H, 32 GB RAM, GTX970M laptop), though small computation is needed for prediction (<0.02 ms @ Intel i7 4720H, 32 GB RAM, GTX970M laptop). However, the envisaged significant expansion of machine and network processing abilities will facilitate increased usage of the proposed method.

## 6. Conclusions

A novel framework based on time-frequency representation and patient-specific DL architectural model for heartbeat classification is proposed. The model performance was validated by evaluation on MIT-BIH arrhythmia database. The results confirmed an overall superior performance with an accuracy of 97.5%. Superior classification results have been achieved by using one encoder layer of SDA with 1024 neurons and one softmax-formed DNN model. The advantages of the proposed framework include its automatic feature extraction, patient-adaptive nature, and low classification error. The proposed patient-specific DNN classifier is simple and effective. Therefore, it is a potential choice for individual automatic heartbeat classification used in WBSN-enabled ECG monitoring.

## Figures and Tables

**Table 1:** Heartbeat classes given by the MIT-BIH database along with the regrouping defined by the AAMI standard [10, 28].

MIT-BIH class	MIT-BIH number	AAMI groups	Number of samples
Normal beat	1	N: beats not found in the classes S, V, F, and Q	90631
Left bundle branch block beat	3		
Right bundle branch block beat	2		
Atrial escape beats	34		
Nodal (junctional) escape beat	11		
Atrial premature beats	8	S: supraventricular ectopic beats	2781
Aberrated atrial premature beats	4		
Nodal (junctional) premature beats	7		
Supraventricular premature beats	9		
Premature ventricular contraction	5	V: ventricular ectopic beats	7236
Ventricular escape beat	10		
Fusion of ventricular and normal beat	6	F: fusion beats	803
Paced beat	12	Q: paced beats or unclassified beats	8010
Fusion of paced and normal beat	38		
Unclassified beat	13		33

**Table 2:** Performance of deep architectural classifier on testing set with reference (performance is determined by interpatient scenario; all testing sets were used).

	Predicted						
		N	S	V	F	Total	SE (%)
True	N	41873	300	947	810	43930	95.3
	S	1520	282	9	25	1836	15.4
	V	1240	13	1943	23	3219	60.4
	F	376	1	9	2	388	0.5
	Total	45009	596	2908	860	49373	42.9
	PPV (%)	93.0	47.3	66.8	0.2	51.8	Accuracy = 89.3%
Accuracy = (TP_N_ + TP_S_ + TP_V_ + TP_F_)/number of testing heartbeats							

**Table 3:** Performance of deep architectural classifier on testing set with reference (performance is determined by trained personalized classifier, testing set excluding the first 300 heartbeats).

	Predicted						
		N	S	V	F	Total	SE (%)
True	N	37622	68	175	119	37984	99.0
	S	448	1143	7	3	1601	71.4
	V	106	0	2644	85	2835	93.3
	F	52	0	9	292	353	82.7
	Total	38228	1211	2835	499	42773	86.6
	PPV (%)	98.4	94.4	93.3	58.5	86.1	Accuracy = 97.5%
Accuracy = (TP_N_ + TP_S_ + TP_V_ + TP_F_)/number of testing heartbeats							

**Table 4:** Classification metrics compared to the state-of-the-art SVEB and VEB classification (percentage, %).

Methods	SVEB				VEB			
	ACC	SE	PPV	SP	ACC	SE	PPV	SP
Proposed (a)	98.8	71.4	94.4	99.8	99.1	93.3	93.3	99.5
Kiranyaz et al. [15]^∗^	96.4	64.6	62.1	98.6	98.6	95	89.5	98.1
Chazal and Reilly [11]^∗^	95.9	87.7	47.0	96.2	99.4	94.3	96.2	99.7
Jiang and Kong [8]^∗^	96.6	50.6	68.0	98.8	97.7	86.6	89.4	98.9
Ince et al. [7]^∗^	97.3	63.5	53.7	98.3	98.0	84.6	86.7	99.0
Proposed (b)	96.2	15.4	47.3	99.3	95.5	60.4	66.8	97.9
Mar et al. [12]	93.3	83.2	33.5	93.7	97.4	86.8	75.9	98.1
Alvarado et al. [10]	97.0	86.2	56.7	97.5	99.1	92.4	93.4	99.5
Ye et al. [9]	97.4	56.4	55.1	98.6	94.6	84.7	59.5	95.4
Zhang et al. [13]	93.3	79.1	36.0	93.9	98.6	85.5	92.7	99.5

^∗^Patient-specific method: require expert intervention.

(a) indicates the patient-specific heartbeat classification scenario. Classifiers were trained by using the first 300 beats of individual patient.

(b) indicates the interpatient heartbeat classification scenario.

**Table 5:** Classification metrics compared to the state-of-the-art four-class heartbeat recognitions (percentage, %).

Methods	Accuracy	*g*-mean		N		S		V		F	
		SE	PPV	SE	PPV	SE	PPV	SE	PPV	SE	PPV
Proposed (a)	97.5	85.9	84.4	99.0	98.4	71.4	94.4	93.3	93.3	82.7	58.5
Chazal and Reilly [11]^∗^	93.9	87.2	59.8	94.3	99.4	87.7	47.0	94.3	96.2	74.0	29.1
Jiang and Kong [8]^∗^	94.5	62.7	83.8	98.7	96.2	50.6	68.0	86.6	89.4	35.8	84.2
Ince et al. [7]^∗^	93.6	74.5	76.9	97.0	97.0	62.1	56.7	83.4	86.5	61.4	73.4
Proposed (b)	89.3	14.6	16.2	95.3	93.0	15.4	47.3	60.4	66.8	0.5	0.2
Mar et al. [12]	89.0	79.3	45.2	94.2	99.2	86.2	56.7	92.4	93.4	66.4	17.7
Alvarado et al. [10]	93.6	84.0	55.2	94.2	99.2	86.2	56.7	92.4	93.4	66.4	17.7
Ye et al. [9]	88.2	62.6	37.0	90.0	98.2	56.4	55.1	84.7	59.5	35.8	5.8
Zhang et al. [13]	88.3	86.7	46.2	88.9	99.0	79.1	36.0	85.5	92.8	93.8	13.7

^∗^Patient-specific method: require expert intervention.

(a) indicates the patient-specific heartbeat classification scenario. Classifiers were trained by using the first 300 beats of individual patient.

(b) indicates interpatient heartbeat classification scenario.
